# Selective serotonin reuptake inhibitors and bleeding risk in patients undergoing PCI on dual antiplatelet therapy: a retrospective cohort study

**DOI:** 10.1093/ehjcvp/pvag034

**Published:** 2026-05-15

**Authors:** Ishmum Chowdhury, Kuan-Yu Chi, Yu Chang, Pei-Lun Lee, Juan Torrado, F Aaysha Cader, Sridhar Mangalesh, Priyansh Shah, Adir Alper, Chidubem Ezenna, Armin Nouri, Raiza Rossi, Zafer Akman, Abdulla A Damluji, Michael G Nanna

**Affiliations:** Jacobi Medical Center, Albert Einstein College of Medicine, Bronx, NY 10461, USA; Jacobi Medical Center, Albert Einstein College of Medicine, Bronx, NY 10461, USA; Section of Neurosurgery, Department of Surgery, National Cheng Kung University Hospital, Tainan 704, Taiwan; Jacobi Medical Center, Albert Einstein College of Medicine, Bronx, NY 10461, USA; Montefiore Medical Center, Albert Einstein College of Medicine, Bronx, NY 10467, USA; Kettering General Hospital NHS Foundation Trust, Kettering NN16 8UZ, United Kingdom; Jacobi Medical Center, Albert Einstein College of Medicine, Bronx, NY 10461, USA; Jacobi Medical Center, Albert Einstein College of Medicine, Bronx, NY 10461, USA; Jacobi Medical Center, Albert Einstein College of Medicine, Bronx, NY 10461, USA; Department of Medicine, University of Massachusetts-Baystate Medical Center, Springfield, MA 01199, USA; Section of Cardiovascular Medicine, Yale School of Medicine, New Haven, CT 06510, USA; Section of Cardiovascular Medicine, Yale School of Medicine, New Haven, CT 06510, USA; Section of Cardiovascular Medicine, Yale School of Medicine, New Haven, CT 06510, USA; Department of Cardiovascular Medicine, The Cardiovascular Center on Aging, the Cleveland Clinic Foundation, Cleveland, OH 44195, USA; Section of Cardiovascular Medicine, Yale School of Medicine, New Haven, CT 06510, USA

**Keywords:** Percutaneous coronary intervention (PCI), Dual antiplatelet therapy (DAPT), Selective serotonin reuptake inhibitors (SSRI), Bleeding, Intracranial haemorrhage, Blood transfusion

## Abstract

**Aims:**

Mood disorders are highly prevalent among patients with coronary artery disease; however, pharmacologic treatment with selective serotonin reuptake inhibitors (SSRIs) poses potential bleeding concerns, particularly in patients receiving dual antiplatelet therapy (DAPT) after percutaneous coronary intervention (PCI). Despite biologic plausibility and observational evidence linking SSRIs to impaired platelet aggregation, data specific to PCI populations remain limited.

**Methods and results:**

Using the TriNetX Global Collaborative Network, we identified adults (≥18 years) who underwent PCI with 12 months of DAPT (aspirin plus a P2Y_12_ inhibitor) between January 2013 and August 2024. Patients were subdivided into SSRI users and non-users. SSRI users were those who had a prescription record within 12 months prior to PCI and refilled records in both the 0- to 6-month and 6- to 12-month periods following index PCI. SSRI non-users were those who had no SSRI prescriptions within 12 months before and after index PCI. Individuals on anticoagulants, serotonin–norepinephrine reuptake inhibitors, or with recent major bleeding were excluded. Propensity-score matching (1:1) was used to balance baseline characteristics between SSRI users and non-users, and time-to-event outcomes were assessed using Kaplan–Meier and Cox proportional-hazards analyses. The primary endpoint was any major bleeding (intracranial haemorrhage [ICH], gastrointestinal bleeding [GIB], requirement for blood transfusion, or other major bleeding) over 1 year; secondary outcomes included ICH, GIB, transfusion, acute myocardial infarction, stroke/TIA, and all-cause mortality Of 29 973 PCI patients on DAPT, 3847 used SSRIs. After matching, 3023 SSRI users were compared with 3023 non-users. Over 1 year, major bleeding occurred in 14.0% of SSRI users vs. 11.3% of non-users (HR 1.28; 95% CI 1.11–1.48; *P* < 0.001). SSRI use was associated with a significantly higher risk of ICH (1.2% vs. 0.7%; HR 1.73; 95% CI 1.01–2.97; *P* = 0.043) and GIB (7.4% vs. 5.6%; HR 1.34; 95% CI 1.10–1.63; *P* = 0.004). Red blood cell transfusion (4.6% vs. 4.1%; HR 1.15; 95% CI 0.90–1.46; *P* = 0.267), all-cause mortality (4.1% vs. 3.8%; HR 1.08; 95% CI 0.83–1.39; *P* = 0.573), and ischaemic outcomes were comparable between groups. The association was strongest in patients aged ≥65 years, with hypertension, chronic kidney disease, or concomitant NSAID use and was consistent across ACS and non-ACS presentations.

**Conclusion:**

In this large, retrospective, real-world cohort, concomitant SSRI therapy during DAPT was associated with an increased bleeding risk, primarily driven by intracranial and gastrointestinal events, without a corresponding increase in ischaemic outcomes, highlighting the importance of clinical awareness of this pharmacodynamic interaction, particularly in higher-risk subgroups, and underscoring the value of established bleeding prevention strategies in this population.

## Graphical Abstract

**Graphical Abstract:**
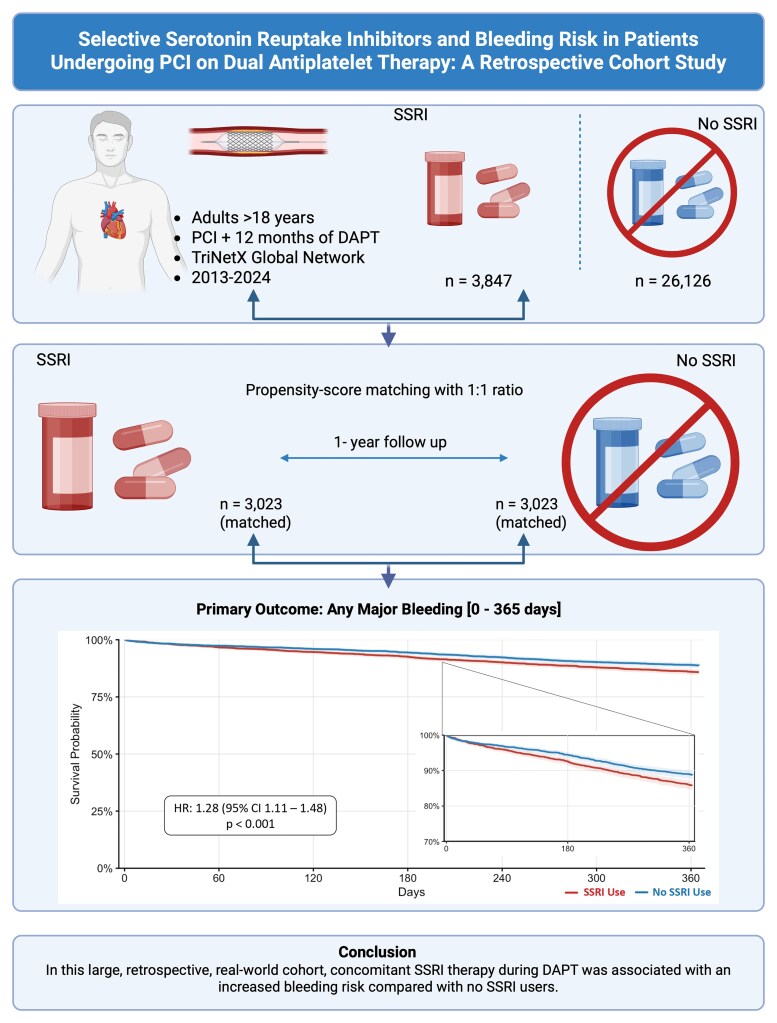
Graphical abstract is created with biorender.com

## Introduction

Mood disorders, particularly depression and anxiety, are highly prevalent in patients with coronary artery disease (CAD).^1^ Up to 40% of patients with CAD have a comorbid mental health condition,^2^ with depression affecting approximately 31% of those with a history of acute coronary syndrome (ACS).^3^ These disorders worsen quality of life, adherence, and functional status,^4,5^ and depression independently predicts recurrent cardiovascular events and mortality, prompting guideline recommendations for routine screening and management.^6^

Selective serotonin reuptake inhibitors (SSRIs) are the mainstay of pharmacologic treatment for mood disorders, including in patients with cardiovascular disease. However, SSRI use is associated with upper gastrointestinal and intracranial bleeding, particularly when combined with nonsteroidal anti-inflammatory drugs (NSAIDs), anticoagulants, or antiplatelets.^7–10^ Concerns are especially relevant when SSRIs are co-prescribed with dual antiplatelet therapy (DAPT), the cornerstone of secondary prevention after percutaneous coronary intervention (PCI), as SSRIs inhibit serotonin reuptake in platelets, depleting intraplatelet serotonin and impairing aggregation.^11–13^ This biological mechanism is supported by observational studies and meta-analyses, with pooled analyses suggesting a 1.4-fold increased risk of major bleeding in SSRI users, particularly among older adults and those with multiple comorbidities, characteristics that are common in PCI populations.^10,14^

However, evidence specific to the bleeding risk of SSRI following PCI and DAPT remains limited. Although Labos and colleagues^10^ found that adding an SSRI to aspirin and clopidogrel significantly increased bleeding risk compared with DAPT alone, other studies found no significant excess risk after adjustment for comorbidities and concomitant medications.^15,16^ A post-hoc analysis of the ROCKET-AF trial (Rivaroxaban vs. Warfarin in Nonvalvular Atrial Fibrillation) found no meaningful increase in major bleeding when SSRIs were combined with anticoagulants.^17^

Given the high prevalence of depression and SSRI use in patients undergoing PCI, clarifying the safety implications of concomitant SSRI and DAPT therapy is clinically important. Leveraging a large multicentre real-world dataset, we aimed to evaluate the influence of concomitant SSRI on bleeding risk after PCI in patients treated with DAPT. We hypothesized that SSRI use would be associated with an increased risk of bleeding complications within 1 year of PCI.

## Methods

### Data source

We conducted a retrospective cohort study using the TriNetX Global Collaborative Network, a federated network of over 150 healthcare organizations (HCOs) aggregating de-identified electronic health records (EHR). The majority of participating HCOs are based in the United States, covering diverse geographic regions, including 39% from the South, 22% from the Northeast, 16% from the Midwest, 13% from the West, and 10% from unspecified areas.^18^ TriNetX standardizes data using common terminologies such as International Classification of Diseases, 10th Revision, Clinical Modification (ICD-10-CM), Current Procedural Terminology (CPT), RxNorm, and Logical Observation Identifiers Names and Codes (LOINC), and allows scalable cohort construction and statistical analysis. Race and ethnicity are self-reported at clinical encounters; missing demographic fields are classified as ‘unknown.’ Mortality is captured from the EHR and linked to external sources where available. Researchers build cohort queries on the TriNetX online portal using these standardized terminologies. Once a cohort is designed, the query is processed by TriNetX's Advanced Analytics Platform, which provides built-in statistical tools for analysis. TriNetX’s data were validated for data completeness,^19^ and there have been numerous studies successfully conducted on comparative effectiveness research using TriNetX,^20–23^ including in the context of cardiovascular disease research.^24–26^

The TriNetX database operates under the Health Insurance Portability and Accountability Act (HIPAA) Privacy Rule as a limited data set, which means that all patient data in the network are de-identified in compliance with HIPAA standards.^18^ All data were fully de-identified in compliance with HIPAA standards, including the removal of direct identifiers such as names, social security numbers, and contact information, ensuring protection of patient privacy. Consequently, the use of de-identified data rendered the study exempt from institutional review board oversight.

### Study population

We identified adult patients (≥18 years) who underwent PCI with subsequent 12-month DAPT therapy between 1 January 2013 and 31 August 2024. DAPT is defined as aspirin with a concomitant P2Y_12_ inhibitor (clopidogrel, prasugrel, or ticagrelor). Both ACS and non-ACS PCI indications were included, and ACS status was recorded as baseline characteristics. To ensure continuous exposure, patients were required to have records of DAPT prescriptions during both the 0- to 6-month and 6- to 12-month periods following index PCI.

Patients were subdivided into two groups: SSRI users and non-users. SSRI users were those who had a prescription for sertraline, paroxetine, fluvoxamine, fluoxetine, citalopram, or escitalopram within 12 months prior to PCI. To ensure continued exposure throughout the DAPT therapy window, SSRI users were also required to have refilled records within 0- to 6-month and 6- to 12-month intervals following index PCI. This three-window requirement was designed to ensure sustained SSRI exposure throughout the entire DAPT period rather than reflecting use at a single time point. SSRI non-users were those who had no SSRI prescriptions within 12 months before and after index PCI. Patients with prior use of anticoagulants, serotonin-norepinephrine reuptake inhibitors (SNRIs), and pregnancy within 1 year prior to index PCI were excluded. SNRIs were excluded rather than used as a comparator, as their additional norepinephrine reuptake inhibition introduces pharmacological heterogeneity incompatible with evaluating a selective serotonin transporter-mediated class effect. To minimize confounding from a high baseline bleeding risk, we excluded patients with end-stage renal disease, dialysis, liver cirrhosis, or any major bleeding event, including intracranial haemorrhage (ICH), gastrointestinal bleeding (GIB), or blood transfusion, within the 12 months preceding index PCI.

### Study outcomes

The primary outcome was any major bleeding, primarily based on the International Society on Thrombosis and Haemostasis (ISTH) definition: a composite of ICH, GIB, blood transfusion, or other major bleeding events (including gross haematuria, haemoperitoneum, haemothorax, haemopericardium, and retroperitoneal haematoma) consistent with the ISTH criteria.^27^ The event of haemoglobin drop ≥2 g/dL was unable to be obtained in TriNetX. The ISTH definition was selected because its component outcomes are all identifiable through ICD-10 coding and structured EHR data, making it the most operationally appropriate validated framework for this database

Secondary outcomes included ICH, GIB, other major bleeding events, and all-cause mortality. Ischaemic outcomes, including acute myocardial infarction (MI) and ischaemic stroke/TIA, were additionally assessed to contextualize the bleeding signal within the broader net clinical benefit framework. We utilized two measures to test the residual confounders in the context of this observational study. First, we set urinary tract infection (UTI) as a falsification endpoint, as it is not expected to be causally related to SSRI exposure. Second, we applied the E-value methodology^28^ to our primary and secondary endpoints by calculating the value of both effect estimates and their lower normal limit. This method estimates the minimum strength of association that an unmeasured confounder would need to have with both the receipt of SSRI and the risk of outcomes to negate the statistically significant effects observed in our study, especially when residual confounding is a potential concern in an observational study.

### Statistical analysis

To reduce confounding, we conducted 1:1 greedy nearest-neighbour propensity-score (PS) matching without replacement, using a calipre width of 0.1 of the standard deviation of the logit of the PS. Covariates, collected from 1 day to 12 months prior to index PCI, included demographics (age, sex, and race/ethnicity), cardiovascular risk factors and comorbidities (hypertension, diabetes mellitus, chronic kidney disease, ischaemic heart disease, prior MI, unstable angina, heart failure, prior stroke/TIA, anaemia, liver disease, substance use disorders, and alcohol use disorder), mood disorders and other indications for SSRI therapy (depression, anxiety, bipolar disorder, post-traumatic stress disorder, obsessive compulsive disorder, fibromyalgia, and perimenopausal symptoms), clinical presentation (ACS vs. non-ACS), medications (antihypertensives, lipid-lowering therapy, antidiabetics, NSAIDs, and gastroprotective agents), laboratory/physiologic measures (haemoglobin, platelets, creatinine, eGFR, LDL cholesterol, albumin, INR, left ventricular ejection fraction, and body mass index), and healthcare utilization (ambulatory visits). Exact ICD-10, RxNorm, ATC, and LOINC codes are provided in Supplementary material online, *Table S1*. Differences in baseline characteristics between SSRI users and non-users were assessed using standardized mean differences (SMD), with a SMD <0.1 considered a balanced distribution of covariates between the two arms. [*Figure 2*] Missing covariate data were not imputed, as the TriNetX platform does not support multiple imputation; variables with incomplete capture were handled as available-case data.

Baseline characteristics were summarized as means with standard deviations for continuous variables and counts with percentages for categorical variables. Between-group differences before matching were assessed using *t*-tests (continuous variables) and χ^2^ tests (categorical variables).

Time-to-event outcomes were analysed using Kaplan–Meier survival curves with log-rank tests. Hazard ratios (HRs) and 95% confidence intervals (CIs) were estimated using Cox proportional-hazards models. A pre-specified landmark analysis was performed at 30 days to differentiate early periprocedural bleeding from late DAPT-related bleeding events. Three pre-specified sensitivity analyses were performed: first, a 2-year follow-up analysis to assess the robustness and durability of findings beyond the primary 1-year endpoint; second, a restriction to clopidogrel-based DAPT users from January 2018 onwards to address potential temporal and pharmacological confounding related to the evolving shift toward more potent P2Y12 inhibitors over the study period, with January 2018 selected to allow sufficient time for the 2017 ESC Focused Update on Dual Antiplatelet Therapy to be adopted into clinical practice^29^; and third, a restriction to new SSRI users only, defined as patients with their first SSRI prescription within 12 months prior to index PCI, to minimize depletion-of-susceptible bias inherent in prevalent user designs. Pre-specified subgroup analyses included age (<65 vs. ≥65 years), sex, diabetes, hypertension, CKD, clinical presentation (ACS vs. non-ACS), and NSAID use.

All analyses were conducted on the TriNetX platform, with supplemental processing performed in *R (version 4.4.0; R Foundation for Statistical Computing).* The following R packages were used: *tidyverse* (data wrangling and visualization), *survival* (Kaplan-Meier and Cox regression), *ggplot2* (plotting), *tableone* (baseline characteristics and SMDs), *patchwork* and *gridExtra* (figure assembly), and *gt* (table formatting). Statistical significance was defined as a two-sided *P* < 0.05.

## Results

### Baseline characteristics

**Figure 1:**
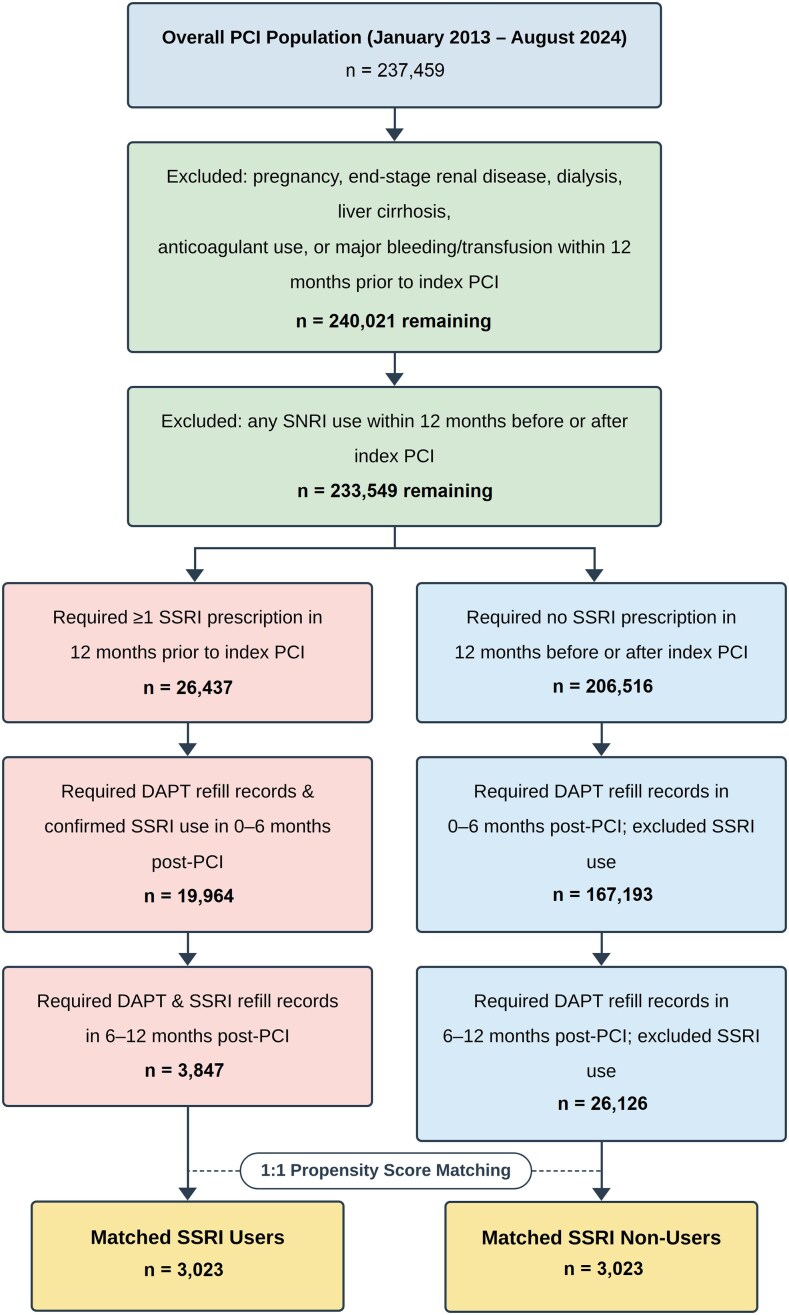
Flow diagram of cohort selection and propensity-score matching of SSRI users and non-users undergoing PCI on DAPT.

A total of 29 973 patients (SSRI users = 3847; non-users = 26 126) undergoing PCI with DAPT between January 2013 and August 2024 were identified. [*Figure 1*].

Before PS matching, SSRI users differed substantially from non-users across demographic and clinical variables. SSRI users were more frequently female (47.9% vs. 28.7%) and White (85.3% vs. 73.0%), while less frequently Black/African American (8.7% vs. 13.8%) or Asian (0.8% vs. 4.5%). They had a markedly higher prevalence of psychiatric conditions such as depressive episode (46.1% vs. 4.1%), major depressive disorder (9.5% vs. 0.7%), anxiety disorder (40.3% vs. 7.0%), and bipolar disorder (3.8% vs. 0.9%). SSRI users also had a higher prevalence of unstable angina (19.1% vs. 11.6%) and MI (36.1% vs. 28.5%). Additionally, they carried a higher burden of comorbidities, including hypertension (80.7% vs. 62.2%), diabetes (50.7% vs. 32.3%), CKD (21.1% vs. 13.3%), heart failure (36.0% vs. 22.7%), and hyperlipidaemia (68.1% vs. 48.4%). These were further reflected by significantly higher prescribed beta-blockers (69.4% vs. 50.2%), ACE inhibitors (34.9% vs. 24.7%), statins (73.7% vs. 53.1%), insulin (39.5% vs. 21.6%), NSAIDs (10% vs. 8%), and proton pump inhibitors (45.6% vs. 24.6%) among SSRI users. SSRI users also had a higher rate of ambulatory visits prior to index PCI (90.6% vs. 76.9%). [*Table 1*]

**Table 1:** Baseline characteristics of patients undergoing PCI on DAPT by SSRI use before and after propensity-score matching

Characteristic	Before PS matching			After PS matching		
	SSRI (n = 3847)	No SSRI (n = 26 126)	SMD	SSRI (n = 3023)	No SSRI (n = 3023)	SMD
**Demographics**						
Age, mean (SD), years	64.6 (11.5)	64.8 (12.2)	0.017	65.0 (11.5)	65.0 (11.9)	<0.001
**Sex**						
Male	2003 (52.1%)	18 619 (71.3%)	0.403	1686 (55.8%)	1598 (52.9%)	0.058
Female	1843 (47.9%)	7503 (28.7%)	0.403	1336 (44.2%)	1425 (47.1%)	0.059
**Race and Ethnicity**						
White	3280 (85.3%)	19 081 (73.0%)	0.304	2566 (84.9%)	2557 (84.6%)	0.008
Black or African American	336 (8.7%)	3613 (13.8%)	0.162	266 (8.8%)	284 (9.4%)	0.021
Hispanic or Latino	138 (3.6%)	1329 (5.1%)	0.074	118 (3.9%)	108 (3.6%)	0.017
Asian	29 (0.8%)	1168 (4.5%)	0.235	25 (0.8%)	20 (0.7%)	0.019
**Baseline Comorbidities**						
Primary hypertension	3105 (80.7%)	16 240 (62.2%)	0.420	2357 (78.0%)	2374 (78.5%)	0.014
Hyperlipidemia	2620 (68.1%)	12 656 (48.4%)	0.407	1970 (65.2%)	1992 (65.9%)	0.015
Type 2 diabetes mellitus	1950 (50.7%)	8448 (32.3%)	0.379	1425 (47.1%)	1469 (48.6%)	0.029
Chronic Kidney Disease	813 (21.1%)	3472 (13.3%)	0.209	584 (19.3%)	583 (19.3%)	0.001
History of ischemic stroke	289 (7.5%)	848 (3.2%)	0.190	190 (6.3%)	203 (6.7%)	0.017
**Cardiovascular History**						
Chronic ischemic cardiac disease	3019 (78.5%)	16 136 (61.8%)	0.371	2307 (76.3%)	2325 (76.9%)	0.014
Unstable Angina	735 (19.1%)	3025 (11.6%)	0.210	527 (17.4%)	558 (18.5%)	0.027
Acute myocardial infarction	1390 (36.1%)	7454 (28.5%)	0.163	1042 (34.5%)	1024 (33.9%)	0.013
Heart failure	1383 (36.0%)	5925 (22.7%)	0.295	1004 (33.2%)	1004 (33.2%)	<0.001
**Psychiatric History & Other SSRI Indications**						
Depressive episode	1772 (46.1%)	1076 (4.1%)	1.105	1007 (33.3%)	980 (32.4%)	0.019
Major depressive disorder	366 (9.5%)	170 (0.7%)	0.412	189 (6.3%)	146 (4.8%)	0.062
Anxiety disorder	1549 (40.3%)	1831 (7.0%)	0.851	971 (32.1%)	1005 (33.2%)	0.024
Bipolar disorder	146 (3.8%)	240 (0.9%)	0.190	96 (3.2%)	116 (3.8%)	0.036
PTSD	118 (3.1%)	135 (0.5%)	0.193	77 (2.5%)	73 (2.4%)	0.009
Alcohol Use Disorder	177 (4.6%)	724 (2.8%)	0.097	118 (3.9%)	131 (4.3%)	0.022
Perimenopausal symptoms	89 (2.3%)	179 (0.7%)	0.134	49 (1.6%)	58 (1.9%)	0.023
Fibromyalgia	137 (3.6%)	250 (1.0%)	0.176	90 (3.0%)	94 (3.1%)	0.008
**Medications**						
Statins	2834 (73.7%)	13 882 (53.1%)	0.436	2125 (70.3%)	2141 (70.8%)	0.012
Beta-blockers	2670 (69.4%)	13 128 (50.2%)	0.398	2011 (66.5%)	2055 (68.0%)	0.031
Calcium channel blockers	1502 (39.0%)	7304 (27.96%)	0.237	1100 (36.4%)	1123 (37.1%)	0.016
ACE inhibitor	1344 (34.9%)	6456 (24.7%)	0.225	1004 (33.2%)	1026 (33.9%)	0.015
ARB	963 (25.0%)	4617 (17.7%)	0.180	709 (23.5%)	707 (23.4%)	0.002
Thiazides	694 (18.0%)	3599 (13.8%)	0.117	528 (17.5%)	520 (17.2%)	0.007
Insulin	1518 (39.5%)	5645 (21.6%)	0.395	1083 (35.8%)	1115 (36.9%)	0.022
Metformin	740 (19.2%)	3130 (12.0%)	0.201	532 (17.6%)	573 (19.0%)	0.035
SGLT2 inhibitors	289 (7.5%)	1136 (4.3%)	0.134	208 (6.9%)	202 (6.7%)	0.008
DPP-4 inhibitors	173 (4.5%)	709 (2.7%)	0.096	123 (4.1%)	137 (4.5%)	0.023
Ibuprofen	353 (9.2%)	1535 (5.9%)	0.125	236 (7.8%)	260 (8.6%)	0.029
Naproxen	163 (4.2%)	726 (2.8%)	0.079	118 (3.9%)	124 (4.1%)	0.010
Ketorolac	439 (11.4%)	1614 (6.2%)	0.186	301 (10.0%)	290 (9.6%)	0.012
Diclofenac	231 (6.0%)	790 (3.0%)	0.144	161 (5.3%)	159 (5.3%)	0.003
**Laboratory Values, mean (SD)**						
BMI (kg/m^2^)	31.5 (7.1)	30.1 (6.3)	0.201	31.3 (6.9)	30.6 (7.1)	0.095
Serum Creatinine (mg/dL)	1.04 (0.36)	1.08 (2.15)	0.028	1.04 (0.36)	1.03 (0.42)	0.030
LVEF (%)	51.2 (14.9)	50.9 (14.8)	0.015	52.4 (14.0)	51.9 (16.4)	0.034
LDL cholesterol (mg/dL)	90.2 (43.8)	96.2 (42.7)	0.138	90.1 (43.9)	94.0 (42.6)	0.091
eGFR (mL/min/1.73 m^2^)	70.9 (25.3)	73.5 (24.9)	0.104	71.1 (25.2)	71.9 (26.1)	0.033
Hemoglobin (g/dL)	12.9 (1.9)	13.5 (2.0)	0.284	13.0 (1.9)	13.1 (2.0)	0.083
Platelets (×10^3^/µL)	234.5 (75.0)	231.0 (73.2)	0.048	232.7 (74.4)	238.1 (75.6)	0.073
Albumin (g/dL)	3.87 (0.50)	3.95 (0.50)	0.155	3.87 (0.49)	3.91 (0.50)	0.083
**Healthcare Utilization**						
Ambulatory visits	3484 (90.6%)	20 101 (76.9%)	0.376	2685 (88.8%)	2690 (89.0%)	0.005

**Figure 2:**
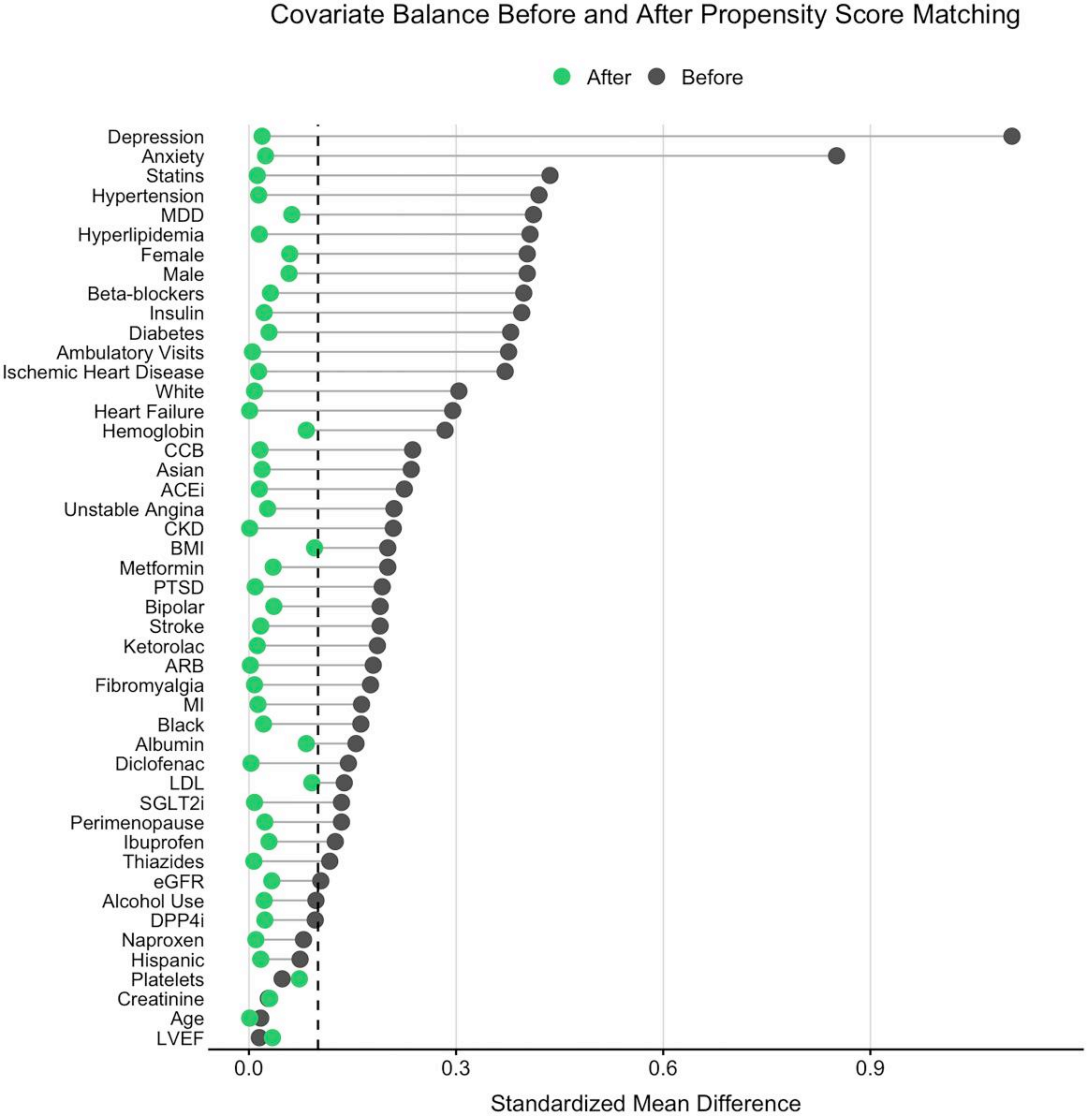
Covariate balance plot showing standardized mean differences before and after propensity score matching. The dashed line indicates the SMD = 0.1 balance threshold.

After PS matching, 3023 SSRI users were matched to 3023 non-users with balanced baseline characteristics across all covariates, as illustrated in the covariate balance plot. [*Figure 2*]

#### Primary outcome

**Figure 3:**
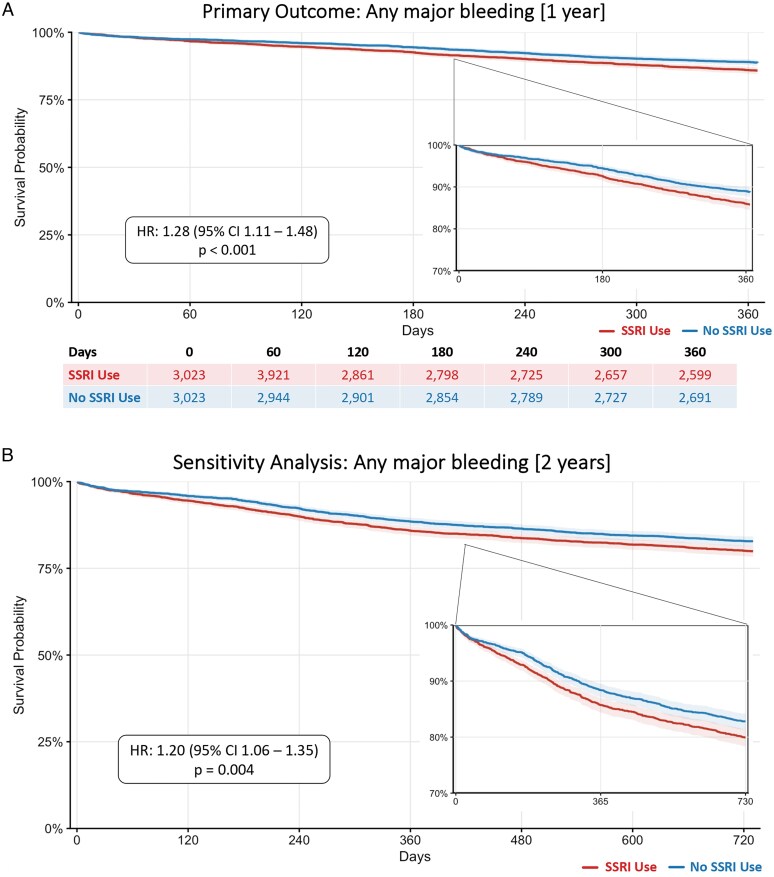
(*A*) Kaplan–Meier estimates of event-free survival for major bleeding over 1 year after PCI in propensity-score–matched cohorts. (*B*) Kaplan–Meier estimates of event-free survival for major bleeding over 2 years after PCI in propensity-score–matched cohorts.

Over the 1-year follow-up, major bleeding occurred in 423 patients (14.0%) in the SSRI cohort compared with 338 patients (11.3%) in the matched control cohort (HR 1.28; 95% CI 1.11–1.48; *P* < 0.001; E-value = 1.88). The survival curves remained largely superimposed during the initial 30 days, after which they began to diverge, with the gap widening steadily over the remainder of the follow-up [*Figure 3A*]. In the 2-year sensitivity analysis of the matched cohort, any major bleeding occurred in 20.1% of SSRI users compared with 17.2% in the control (HR 1.20; 95% CI 1.06–1.35; *P* = 0.004; E-value = 1.69) [*Figure 3B*]. In a sensitivity analysis restricted to clopidogrel-based DAPT users from January 2018 onwards, major bleeding occurred in 15.5% of SSRI users compared with 12.0% of non-users (HR 1.33; 95% CI 1.08–1.63; *P* = 0.006; E-value = 1.99). In a further sensitivity analysis restricted to new SSRI users only, major bleeding occurred in 13.2% of SSRI users compared with 10.2% of non-users (HR 1.32; 95% CI 1.05–1.67; *P* = 0.019; E-value = 1.97). In the landmark analysis, major bleeding was comparable between groups in the first 30 days (1.9% vs. 2.1%; HR 0.93; 95% CI 0.65–1.34; *P* = 0.710), while a significant excess risk was observed between 31–365 days in SSRI users (12.8% vs. 9.6%; HR 1.36; 95% CI 1.17–1.59; *P* < 0.001).

**Figure 4:**
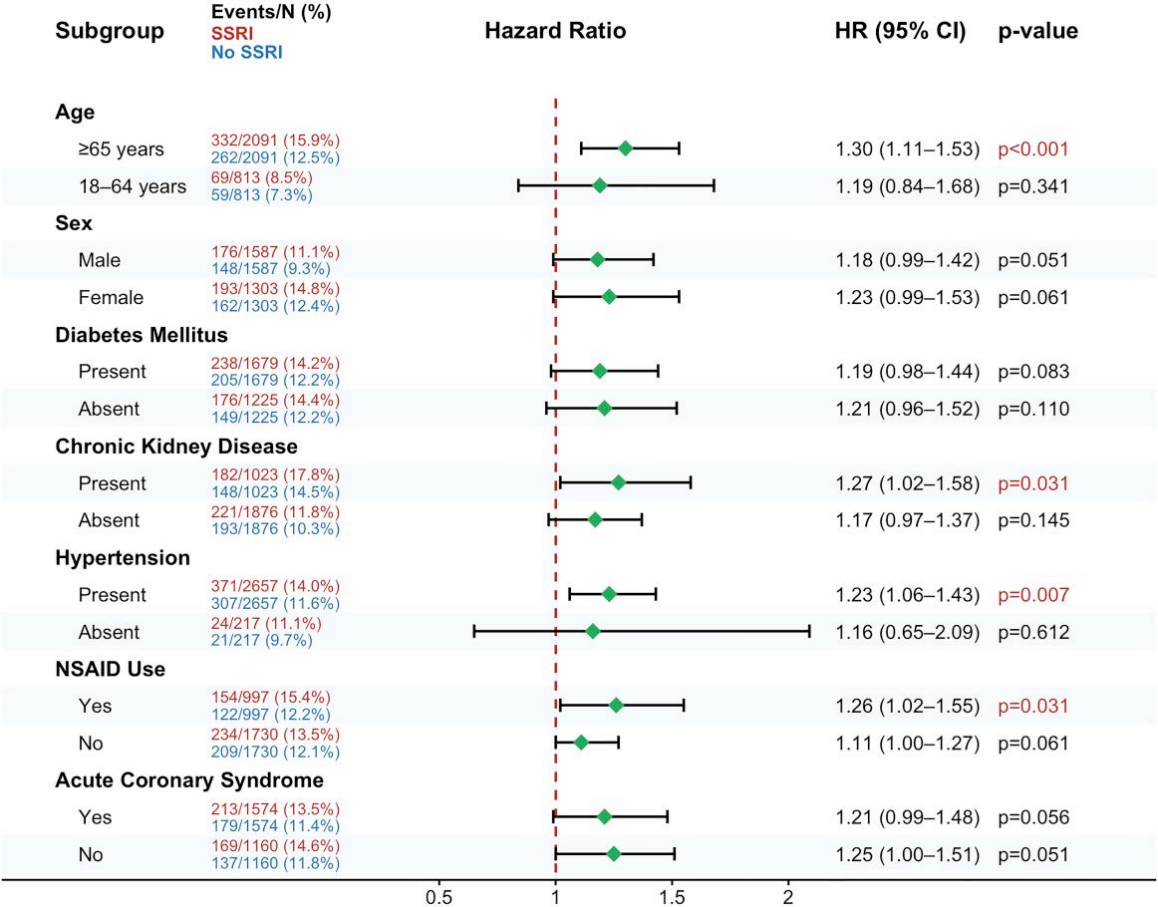
Subgroup analyses of bleeding outcomes by SSRI use after PCI in propensity-matched cohorts.

Prespecified subgroup analyses demonstrated that the increased risk of major bleeding associated with SSRI use was generally consistent across key clinical subgroups [*Figure 4*]. The association was particularly pronounced among patients aged ≥65 years (HR 1.30; 95% CI 1.11–1.53; *P* < 0.001), those with hypertension (HR 1.23; 95% CI 1.06–1.43; *P* = 0.007), CKD (HR 1.27; 95% CI 1.02–1.58; *P* = 0.031), and concomitant NSAID use (HR 1.26; 95% CI 1.02–1.55; *P* = 0.031). While neither ACS subgroup reached conventional statistical significance in isolation, the association was directionally consistent regardless of ACS presentation (ACS present: HR 1.21; 95% CI 0.99–1.48; *P* = 0.056; ACS absent: HR 1.25; 95% CI 1.00–1.51; *P* = 0.051).

#### Secondary outcomes

**Figure 5:**
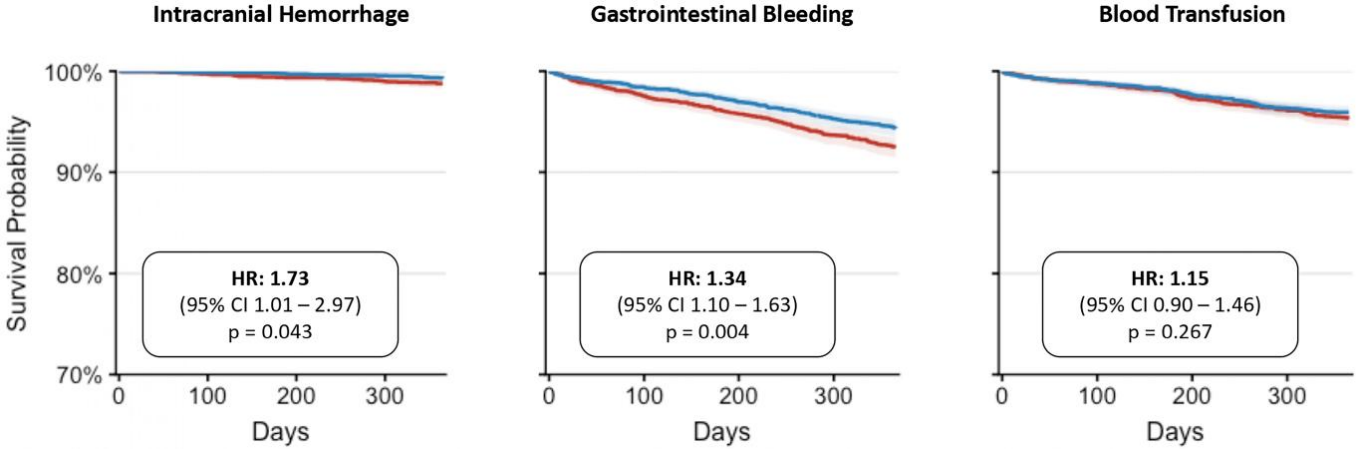
Kaplan–Meier estimates of secondary outcomes over 1 year after PCI by SSRI use in propensity-score–matched cohorts. *(Note: the lower range of the y-axis is truncated at 70% to enhance visualization of between-group differences.)*

SSRI use was associated with a significantly higher risk of ICH (1.2% vs. 0.7%; HR 1.73; 95% CI 1.01–2.97; *P* = 0.043; E-value = 2.85) and GIB (7.4% vs. 5.6%; HR 1.34; 95% CI 1.10–1.63; *P* = 0.004; E-value = 2.02). Red blood cell transfusion was not significantly increased in SSRI users (4.6% vs. 4.1%; HR 1.15; 95% CI 0.90–1.46; *P* = 0.267). [*Figure 5*] All-cause mortality occurred in 124 (4.1%) SSRI users and 116 (3.8%) non-users (HR 1.08; 95% CI 0.83–1.39; *P* = 0.573), with no significant difference between groups. Ischaemic outcomes were comparable between groups, with no significant difference in acute MI (HR 0.97; 95% CI 0.91–1.05; *P* = 0.504) or ischaemic stroke/TIA (HR 1.14; 95% CI 0.98–1.32; *P* = 0.078).

For the falsification endpoint, there was no difference in urinary tract infection between SSRI users and non-users (13.1% vs. 11.8%; HR 1.12; 95% CI 0.97–1.29; *P* = 0.116).

## Discussion

In this large, multicentre propensity-matched analysis of all-comer patients undergoing PCI, concomitant SSRI therapy during DAPT was associated with an increased risk of major bleeding, primarily driven by ICH and GIB. While the absolute event rates appear higher than those reported in contemporary adjudicated trials,^30,31^ this likely reflects EHR-based coding, whereby any qualifying ICD-10 diagnosis is captured irrespective of symptom documentation, unlike adjudicated endpoints. The landmark analysis further demonstrated that bleeding risk was comparable in the first 30 days but diverged significantly thereafter, suggesting that the excess bleeding risk associated with SSRI use is concentrated during the sustained DAPT exposure period rather than driven by early periprocedural factors. Notably, survival curves in the 2-year sensitivity analysis appeared parallel after the 1-year landmark, consistent with the excess bleeding risk attenuating following DAPT discontinuation, consistent with the hypothesis that the synergistic pharmacodynamic interaction between SSRI-mediated SERT inhibition and P2Y12 blockade may attenuate upon transition to single antiplatelet therapy. Ischaemic outcomes were comparable between groups, suggesting that the observed signal is specific to the bleeding domain. These findings reinforce the biological plausibility linking serotonin reuptake inhibition with impaired platelet aggregation and highlight a clinically important intersection between psychiatric and cardiovascular care, with important implications for secondary prevention strategies. The consistency of findings across three pre-specified sensitivity analyses, including a restriction to clopidogrel-based DAPT users from 2018 onwards and a new-user design, further supports the robustness of the primary association.

SSRIs deplete intraplatelet serotonin stores by inhibiting SERT, attenuating serotonin-mediated P2Y12 amplification, and impairing platelet aggregation; when combined with DAPT, which directly inhibits P2Y12, it may create a mechanistically synergistic dual pharmacodynamic burden.^32,33^ While this may normalize hyperreactivity observed in patients with mood disorders and CAD,^34,35^ it also compromises haemostasis, conferring bleeding risk.^36^ This is supported by prior observational studies reporting elevated bleeding rates with SSRIs on DAPT,^10^ and a recent meta-analysis confirming elevated GIB risk across all commonly used SSRIs (OR 1.31–1.38).^37^ However, the literature is not uniform, and studies in cardiac surgical populations, particularly following CABG, have reported neutral associations between SSRI use and perioperative bleeding,^38,39^ suggesting that the pharmacodynamic interaction between SSRIs and antiplatelet agents may be context- and duration-dependent. None of these prior studies were specific to PCI patients on long-term DAPT, and our analysis uniquely addresses this gap. SSRI-associated bleeding risk was most pronounced in older patients and those with hypertension, CKD, or concomitant NSAID use, populations inherently predisposed to impaired haemostasis, where age-related vascular fragility, hypertensive small vessel disease, uraemic platelet dysfunction, and prostaglandin-mediated gastroprotection inhibition may each compound the pharmacodynamic vulnerability conferred by SSRI-DAPT co-exposure.^40–42^ The consistency of this association across ACS and non-ACS presentations, in a cohort where antithrombotic intensity was homogenized by design through the 12-month DAPT requirement, further supports a pharmacodynamic rather than antithrombotic intensity-driven mechanism.

Mood disorders are well-established prognostic factors after ACS. The MIND-IT trial demonstrated that psychiatric symptom burden is independently associated with impaired cardiac recovery.^43^ Proposed mechanisms include inflammatory activation, autonomic and endothelial dysfunction, abnormal platelet signalling, neuroendocrine dysregulation, and behavioural factors such as reduced adherence and inactivity.^44–48^ Thus, mood disorders not only complicate recovery but also worsen cardiovascular prognosis, making their treatment essential after PCI. Whether incorporating SSRI exposure into established bleeding risk frameworks such as PRECISE-DAPT^49^ and ARC-HBR^50^ could further refine bleeding risk stratification in this population remains an interesting question for future investigation. Current guidelines acknowledge SSRI safety in CAD^51^ but provide no specific recommendations for PCI patients on DAPT. Our findings suggest that concomitant SSRI use may represent an additional contributor to bleeding risk in this population, particularly in patients already at high baseline bleeding risk. In this context, clinicians may consider the implementation of well-established bleeding mitigation strategies, such as gastrointestinal protection with proton pump inhibitors alongside optimization of other modifiable bleeding risk factors such as blood pressure control.^52^

## Limitations

Our study has several limitations. As with any observational analysis, residual and unmeasured confounding cannot be fully excluded despite rigorous propensity score matching and comparable falsification endpoints; the modest E-values for several endpoints suggest that the observed associations may be sensitive to unmeasured confounders, and causal inference should be interpreted cautiously. Bleeding outcomes were identified through coded diagnoses entered at the treating physician’s discretion rather than adjudicated events, which may introduce misclassification bias. The BARC criteria could not be applied, given the absence of granular procedural variables in TriNetX; the ISTH framework was therefore selected as the most operationally feasible validated definition, although its anatomical coding approach, whereby any qualifying ICD-10 diagnosis is captured regardless of symptom documentation, likely yields higher absolute event rates than adjudicated trial endpoints. SSRI dose, duration, and adherence could not be evaluated from dispensing records alone, and missing covariate data were handled as available-case data, given the platform constraints. The 12-month DAPT requirement, while necessary to create a homogeneous exposure window aligned with SSRI refill verification periods, may limit generalizability to contemporary CCS patients managed with shorter DAPT durations per current ESC guidelines.^53^ Exclusion of patients with recent major bleeding, end-stage renal disease, dialysis, liver cirrhosis, and anticoagulant use was necessary to prevent outcome misclassification, but it limits generalizability to the highest-risk populations. Several analytic limitations are attributable to the TriNetX platform, including the inability to perform stratified Cox regression accounting for the matched pair structure, which may introduce imprecision in the standard error estimates, as well as the inability to perform Fine-Gray competing risk models, full multivariable model coefficient reporting, and formal interaction testing for subgroup analysis.

## Conclusions

In a large, real-world PCI population, concomitant SSRI therapy during DAPT was associated with a modestly increased bleeding risk, primarily driven by intracranial and gastrointestinal events, without a corresponding increase in ischaemic outcomes. These findings highlight an important pharmacodynamic interaction between SSRIs and DAPT that warrants clinical awareness, particularly in higher-risk subgroups such as older patients, those with CKD, hypertension, or concomitant NSAID use. Practical strategies to help mitigate this risk may include careful bleeding risk assessment, thoughtful antidepressant selection, and close collaboration between cardiology and psychiatry teams. Whether SSRI use should be formally incorporated into established bleeding risk frameworks represents an important question requiring dedicated prospective investigation. Pending such evidence, clinicians should remain vigilant to the potential additive bleeding risk of concomitant SSRI use during DAPT, and implementation of well-established bleeding prevention strategies, including gastrointestinal protection with proton pump inhibitors and blood pressure optimization, should be considered in higher-risk patients.

## Supplementary Material

pvag034_Supplementary_Data

## Data Availability

The data underlying this study were accessed through the TriNetX Global Collaborative Network, a federated network of de-identified electronic health records. The data are not publicly available as they are proprietary to the contributing healthcare organizations and accessed under a data use agreement. All ICD-10-CM, RxNorm, CPT, ATC, and LOINC codes used to define cohort inclusion and exclusion criteria, exposures, covariates, and outcomes in the TriNetX query are provided in full in Supplementary material online, *Table S1*. Researchers interested in replicating this study may apply for access to the TriNetX platform at www.trinetx.com. Additional analytic details are available from the corresponding author upon request.
