# Supplementary material for: Selective serotonin reuptake inhibitors and bleeding risk in patients undergoing PCI on dual antiplatelet therapy: a retrospective cohort study
Source: Eur Heart J Cardiovasc Pharmacother. 2026 May 15;12(4):331–42. doi: 10.1093/ehjcvp/pvag034 (PMC13367246; doi:10.1093/ehjcvp/pvag034)
Supplement: pvag034_Supplementary_Data [file pvag034_supplementary_data.zip › Supplementary File 2.docx]

**Supplementary Methods**

**Study Protocol**

Selective Serotonin Reuptake Inhibitors and Bleeding Risk in Patients Undergoing PCI on Dual Antiplatelet Therapy: A Retrospective Cohort Study

**1. Study Design and Setting**

This was a retrospective cohort study conducted using the TriNetX Global Collaborative Network, a federated network of over 150 healthcare organizations (HCOs) aggregating de-identified electronic health records (EHR) from diverse geographic regions across the United States and internationally. Data were standardized using ICD-10-CM, CPT, RxNorm, ATC, and LOINC terminologies. All patient data were de-identified in compliance with HIPAA standards, and the study was exempt from institutional review board oversight.

The study period spanned January 1, 2013 through August 31, 2024.

**2. Study Population**

**2.1 Inclusion Criteria**

- Adults aged ≥18 years
- Underwent percutaneous coronary intervention (PCI) between January 1, 2013 and August 31, 2024, identified using CPT codes (1021163, 1021164, 1021165, 1021166, 1021167, 1021168, 92941)
- Received dual antiplatelet therapy (DAPT), defined as aspirin (RxNorm: 1191) plus a P2Y12 inhibitor (clopidogrel RxNorm: 32968; ticagrelor RxNorm: 1116632; prasugrel RxNorm: 613391), with confirmed prescription records in both the 0–6 month and 6–12 month periods following index PCI
- Both ACS and non-ACS PCI indications were included

**2.2 Exclusion Criteria**

- Anticoagulant use within 12 months prior to index PCI (warfarin, apixaban, rivaroxaban, dabigatran, edoxaban)
- Any serotonin-norepinephrine reuptake inhibitor (SNRI) use within 12 months before or after index PCI (venlafaxine, desvenlafaxine, duloxetine, milnacipran, levomilnacipran)
- Pregnancy within 12 months prior to index PCI
- End-stage renal disease or dialysis dependence
- Liver cirrhosis
- Any major bleeding event within 12 months prior to index PCI, including intracranial hemorrhage (ICH), gastrointestinal bleeding (GIB), or blood transfusion

These exclusions were applied to minimize confounding from high baseline bleeding risk and to prevent misclassification of outcomes occurring prior to the index date.

**3. Exposure Definition**

**3.1 SSRI Users (Cohort 1)**

Patients were classified as SSRI users if they had a prescription record for any of the following agents within 12 months prior to index PCI: sertraline (RxNorm: 36437), paroxetine (RxNorm: 32937), fluvoxamine (RxNorm: 42355), fluoxetine (RxNorm: 4493), citalopram (RxNorm: 2556), or escitalopram (RxNorm: 321988).

To ensure sustained exposure throughout the DAPT period, SSRI users were additionally required to have confirmed refill records in both the 0–6 month and 6–12 month periods following index PCI. This three-window requirement was designed to capture persistent SSRI exposure throughout the entire DAPT window rather than a single time point.

**3.2 SSRI Non-Users (Cohort 2)**

Patients were classified as SSRI non-users if they had no prescription records for any of the six SSRIs listed above within 12 months before or after index PCI.

**4. Outcome Definitions**

**4.1 Primary Outcome**

Any major bleeding over 1-year follow-up, defined as a composite of intracranial hemorrhage (ICH), gastrointestinal bleeding (GIB), blood transfusion, or other major bleeding events (gross hematuria, hemoperitoneum, hemothorax, hemopericardium, retroperitoneal hematoma), consistent with the International Society on Thrombosis and Hemostasis (ISTH) framework. Outcomes were identified through ICD-10-CM and ICD-10-PCS codes. The hemoglobin drop criterion (≥2 g/dL) required by the formal ISTH definition could not be individually linked to bleeding episodes within TriNetX and was therefore not applied.

**4.2 Secondary Outcomes**

- Intracranial hemorrhage (ICH): ICD-10-CM I60, I61, I62
- Gastrointestinal bleeding (GIB): ICD-10-CM K92.0, K92.1, K92.2, K28.0, K28.4
- Red blood cell transfusion: ICD-10-PCS 30233N0, 30233N1, 30230N1, 30243N0
- All-cause mortality: death record in TriNetX EHR
- Acute myocardial infarction: ICD-10-CM I21, I22
- Ischemic stroke/TIA: ICD-10-CM I63, G45

**4.3 Falsification Endpoint**

Urinary tract infection (ICD-10-CM N39.0) was used as a falsification endpoint, as it has no plausible pharmacodynamic relationship to SSRI exposure. A significant association would suggest residual confounding or differential healthcare contact rather than a true pharmacologic effect.

**4.4 Sensitivity Analysis for Unmeasured Confounding**

E-values were calculated for all statistically significant outcomes using the method of VanderWeele and Ding (Ann Intern Med. 2017). The E-value represents the minimum strength of association that an unmeasured confounder would need to have with both SSRI use and the outcome to fully explain the observed association.

**5. Propensity Score Matching**

**5.1 Matching Algorithm**

To reduce confounding by indication and baseline differences between SSRI users and non-users, 1:1 greedy nearest-neighbor propensity score (PS) matching was performed without replacement, using a caliper width of 0.1 standard deviations of the logit of the propensity score.

**5.2 Covariates Included in the PS Model**

Covariates were collected from 1 day to 12 months prior to index PCI and included:

- Demographics: age, sex, race/ethnicity, body mass index, blood pressure
- Cardiovascular risk factors and comorbidities: hypertension, diabetes mellitus, chronic kidney disease, ischemic heart disease, prior MI, unstable angina, heart failure, prior stroke/TIA, anemia, liver disease, substance use disorders, alcohol use disorder
- Psychiatric history and other SSRI indications: depression, anxiety, bipolar disorder, PTSD, OCD, fibromyalgia, perimenopausal symptoms
- Clinical presentation: ACS vs non-ACS
- Concomitant medications: antihypertensives, lipid-lowering therapy, antidiabetics, NSAIDs, gastroprotective agents
- Laboratory/physiologic measures: hemoglobin, platelets, creatinine, eGFR, LDL cholesterol, albumin, INR, left ventricular ejection fraction
- Healthcare utilization: ambulatory visits within 12 months prior to index PCI

**5.3 Covariate Balance Assessment**

Balance between SSRI users and non-users before and after PS matching was assessed using standardized mean differences (SMD). A SMD <0.10 was considered indicative of adequate balance. A covariate balance plot (love plot) illustrating SMDs before and after matching for all covariates is provided as Supplementary Figure 1.

**6. Statistical Analysis**

**6.1 Primary Analysis**

Time-to-event outcomes were analyzed using Kaplan-Meier survival curves with log-rank tests. Hazard ratios (HRs) and 95% confidence intervals (CIs) were estimated using Cox proportional hazards models. All analyses were conducted in the matched cohort.

**6.2 Landmark Analysis**

A pre-specified landmark analysis was performed at 30 days to differentiate early periprocedural bleeding from late DAPT-related bleeding events. Results are reported separately for the 0–30 day and 31–365 day periods.

**6.3 Sensitivity Analysis**

A pre-specified sensitivity analysis was performed using a 2-year follow-up horizon to assess the robustness and durability of findings beyond the primary 1-year endpoint.

An additional sensitivity analysis was performed restricting the cohort to patients receiving clopidogrel-based DAPT only, within the post-2018 era, to address potential temporal confounding related to the evolving shift from clopidogrel to more potent P2Y12 inhibitors over the study period. January 2018 was selected as the index date to allow sufficient time for the 2017 ESC Focused Update on Dual Antiplatelet Therapy to be adopted into clinical practice.

**6.4 Subgroup Analyses**

Pre-specified subgroup analyses were performed for the primary outcome across the following variables:

- Age (<65 vs ≥65 years)
- Sex (male vs female)
- Diabetes mellitus (present vs absent)
- Chronic kidney disease (present vs absent)
- Hypertension (present vs absent)
- Concomitant NSAID use (yes vs no)
- Clinical presentation (ACS vs non-ACS)

**6.5 Software**

All analyses were conducted on the TriNetX Analytics Platform. Supplemental processing and figure generation were performed in R (version 4.4.0; R Foundation for Statistical Computing) using the following packages: tidyverse, survival, ggplot2, tableone, patchwork, gridExtra, and gt. Statistical significance was defined as a two-sided p <0.05.

**6.6 Missing Data**

Missing covariate data were not imputed, as the TriNetX platform does not support multiple imputation. Variables with incomplete capture were handled as available-case data, meaning patients were included in analyses for covariates where data were available.

**Complete ICD-10-CM, RxNorm, CPT, ATC, and LOINC codes used to define all cohort criteria, exposures, covariates, and outcomes are provided in Supplementary Table 1.*
