# Supplementary material for: Selective serotonin reuptake inhibitors and bleeding risk in patients undergoing PCI on dual antiplatelet therapy: a retrospective cohort study
Source: Eur Heart J Cardiovasc Pharmacother. 2026 May 15;12(4):331–42. doi: 10.1093/ehjcvp/pvag034 (PMC13367246; doi:10.1093/ehjcvp/pvag034)
Supplement: pvag034_Supplementary_Data [file pvag034_supplementary_data.zip › Supplementary Table 1.docx]

**Supplementary Table 1. ICD-10-CM, RxNorm, CPT, and LOINC Codes Used in the TriNetX Query**

| Category | Variable/Term | Code(s) | Code System |
| --- | --- | --- | --- |
| **Procedures** | | | |
| PCI | Percutaneous transluminal coronary angioplasty | 1021163 | CPT |
| PCI | PCI with coronary atherectomy | 1021164 | CPT |
| PCI | Intracoronary stent placement with angioplasty | 1021165 | CPT |
| PCI | PCI with atherectomy and stent | 1021166 | CPT |
| PCI | PCI via coronary artery bypass graft | 1021167 | CPT |
| PCI | PCI of chronic total occlusion | 1021168 | CPT |
| PCI | PCI of acute total/subtotal occlusion during MI | 92941 | CPT |
|  |  |  |  |
| **SSRI Medications** | | | |
| SSRI | Citalopram | 2556 | RxNorm |
| SSRI | Escitalopram | 321988 | RxNorm |
| SSRI | Paroxetine | 32937 | RxNorm |
| SSRI | Fluvoxamine | 42355 | RxNorm |
| SSRI | Sertraline | 36437 | RxNorm |
| SSRI | Fluoxetine | 4493 | RxNorm |
|  |  |  |  |
| **Antiplatelet Therapy** | | | |
| DAPT | Aspirin | 1191 | RxNorm |
| DAPT | Clopidogrel | 32968 | RxNorm |
| DAPT | Ticagrelor | 1116632 | RxNorm |
| DAPT | Prasugrel | 613391 | RxNorm |
|  |  |  |  |
| **Exclusions** | | | |
| Anticoagulant Exclusion | Warfarin | 11289 | RxNorm |
| Anticoagulant Exclusion | Apixaban | 1364430 | RxNorm |
| Anticoagulant Exclusion | Rivaroxaban | 1114195 | RxNorm |
| Anticoagulant Exclusion | Dabigatran | 1037042 | RxNorm |
| Anticoagulant Exclusion | Edoxaban | 1599538 | RxNorm |
| SNRI Exclusion | Venlafaxine | 39786 | RxNorm |
| SNRI Exclusion | Desvenlafaxine | 734064 | RxNorm |
| SNRI Exclusion | Duloxetine | 72625 | RxNorm |
| SNRI Exclusion | Milnacipran | 588250 | RxNorm |
| SNRI Exclusion | Levomilnacipran | 1433212 | RxNorm |
| Clinical Exclusion | Pregnancy | 10 | ICD-10-CM |
| Clinical Exclusion | End-stage renal disease | N18.6 | ICD-10-CM |
| Clinical Exclusion | Dependence on renal dialysis | Z99.2 | ICD-10-CM |
| Clinical Exclusion | Fibrosis and cirrhosis of liver | K74 | ICD-10-CM |
| Clinical Exclusion | Nontraumatic subarachnoid hemorrhage | I60 | ICD-10-CM |
| Clinical Exclusion | Nontraumatic intracerebral hemorrhage | I61 | ICD-10-CM |
| Clinical Exclusion | Other nontraumatic intracranial hemorrhage | I62 | ICD-10-CM |
| Clinical Exclusion | Hematemesis | K92.0 | ICD-10-CM |
| Clinical Exclusion | Melena | K92.1 | ICD-10-CM |
| Clinical Exclusion | Gastrointestinal hemorrhage, unspecified | K92.2 | ICD-10-CM |
| Clinical Exclusion | Acute gastrojejunal ulcer with hemorrhage | K28.0 | ICD-10-CM |
| Clinical Exclusion | Chronic gastrojejunal ulcer with hemorrhage | K28.4 | ICD-10-CM |
| Clinical Exclusion | Hematuria | R31 | ICD-10-CM |
| Clinical Exclusion | Hemoperitoneum | K66.1 | ICD-10-CM |
| Clinical Exclusion | Hemopericardium | I31.2 | ICD-10-CM |
| Clinical Exclusion | Retroperitoneal hematoma | K68.3 | ICD-10-CM |
| Clinical Exclusion | Transfusion – autologous RBC, peripheral vein, percutaneous | 30233N0 | ICD-10-PCS |
| Clinical Exclusion | Transfusion – nonautologous RBC, peripheral vein, open | 30230N1 | ICD-10-PCS |
| Clinical Exclusion | Transfusion – nonautologous RBC, peripheral vein, percutaneous | 30233N1 | ICD-10-PCS |
| Clinical Exclusion | Transfusion – autologous RBC, central vein, percutaneous | 30243N0 | ICD-10-PCS |
|  |  |  |  |
| **Baseline Comorbidities** | | | |
| Comorbidity | Primary hypertension | I10 | ICD-10-CM |
| Comorbidity | Type 2 diabetes mellitus | E11 | ICD-10-CM |
| Comorbidity | Hyperlipidemia | E78 | ICD-10-CM |
| Comorbidity | Chronic kidney disease | N18 | ICD-10-CM |
| Comorbidity | Heart failure | I50 | ICD-10-CM |
| Comorbidity | Ischemic heart disease (chronic) | I25 | ICD-10-CM |
| Comorbidity | Acute myocardial infarction | I21, I22 | ICD-10-CM |
| Comorbidity | Unstable angina | I20.0 | ICD-10-CM |
| Comorbidity | History of ischemic stroke | I63 | ICD-10-CM |
| Comorbidity | Liver disease | K70–K77 | ICD-10-CM |
| Comorbidity | Anemia | D50–D64 | ICD-10-CM |
| Comorbidity | Alcohol use disorder | F10 | ICD-10-CM |
| Comorbidity | Substance use disorders | F11–F19 | ICD-10-CM |
| Comorbidity | Depressive episode | F32 | ICD-10-CM |
| Comorbidity | Major depressive disorder | F33 | ICD-10-CM |
| Comorbidity | Anxiety disorder | F40–F41 | ICD-10-CM |
| Comorbidity | Bipolar disorder | F31 | ICD-10-CM |
| Comorbidity | PTSD | F43.1 | ICD-10-CM |
| Comorbidity | OCD | F42.0, F42.2, F42.3, F42.4, F42.8, F42.9 | ICD-10-CM |
| Comorbidity | Fibromyalgia | M79.7 | ICD-10-CM |
| Comorbidity | Perimenopausal symptoms | N95.1 | ICD-10-CM |
|  |  |  |  |
| **Baseline medications** | | | |
| Medication | Beta-blockers | ATC: C07 | ATC |
| Medication | ACE inhibitors | ATC: C09A | ATC |
| Medication | ARBs | ATC: C09C | ATC |
| Medication | Calcium channel blockers | ATC: C08 | ATC |
| Medication | Thiazide diuretics | ATC: C03A | ATC |
| Medication | Statins | ATC: C10AA | ATC |
| Medication | Insulin | ATC: A10A | ATC |
| Medication | Metformin | ATC: A10BA02 | ATC |
| Medication | SGLT2 inhibitors | ATC: A10BK | ATC |
| Medication | DPP-4 inhibitors | ATC: A10BH | ATC |
| Medication | Ibuprofen | 5640 | RxNorm |
| Medication | Naproxen | 7258 | RxNorm |
| Medication | Ketorolac | 35827 | RxNorm |
| Medication | Diclofenac | 3355 | RxNorm |
| Medication | Proton pump inhibitors | ATC: A02BC | ATC |
| Medication | H2 blockers | ATC: A02BA | ATC |
| Medication | Mirtazapine | 15996 | RxNorm |
| Medication | Bupropion | 42347 | RxNorm |
|  |  |  |  |
| **Baseline laboratory and physiological measures** | | | |
| Laboratory | Hemoglobin | 718-7 | LOINC |
| Laboratory | Platelet count | 777-3 | LOINC |
| Laboratory | Serum creatinine | 2160-0 | LOINC |
| Laboratory | eGFR | 62238-1 | LOINC |
| Laboratory | LDL cholesterol | 2089-1 | LOINC |
| Laboratory | Serum albumin | 1751-7 | LOINC |
| Laboratory | INR | 6301-6 | LOINC |
| Laboratory | Left ventricular ejection fraction | 10230-1 | LOINC |
| Laboratory | BMI | 39156-5 | LOINC |
| Laboratory | Systolic blood pressure | 8480-6 | LOINC |
|  |  |  |  |
| **Healthcare Utilization** | | | |
| Healthcare Utilization | Ambulatory Visits | AMB | TriNetX Visit Code |
|  |  |  |  |
| **Primary Outcome** | | | |
| Primary Outcome | Nontraumatic subarachnoid hemorrhage (ICH) | I60 | ICD-10-CM |
| Primary Outcome | Nontraumatic intracerebral hemorrhage (ICH) | I61 | ICD-10-CM |
| Primary Outcome | Other nontraumatic intracranial hemorrhage (ICH) | I62 | ICD-10-CM |
| Primary Outcome | Hematemesis (GIB) | K92.0 | ICD-10-CM |
| Primary Outcome | Melena (GIB) | K92.1 | ICD-10-CM |
| Primary Outcome | Gastrointestinal hemorrhage, unspecified (GIB) | K92.2 | ICD-10-CM |
| Primary Outcome | Acute gastrojejunal ulcer with hemorrhage (GIB) | K28.0 | ICD-10-CM |
| Primary Outcome | Chronic gastrojejunal ulcer with hemorrhage (GIB) | K28.4 | ICD-10-CM |
| Primary Outcome | Hematuria | R31 | ICD-10-CM |
| Primary Outcome | Hemoperitoneum | K66.1 | ICD-10-CM |
| Primary Outcome | Hemopericardium | I31.2 | ICD-10-CM |
| Primary Outcome | Retroperitoneal hematoma | K68.3 | ICD-10-CM |
| Primary Outcome | RBC transfusion – peripheral vein | 30233N0, 30233N1, 30230N1 | ICD-10-PCS |
| Primary Outcome | RBC transfusion – central vein | 30243N0 | ICD-10-PCS |
|  |  |  |  |
| **Secondary Outcomes** | | | |
| Secondary Outcome | Intracranial hemorrhage (ICH) – composite | I60, I61, I62 | ICD-10-CM |
| Secondary Outcome | Gastrointestinal bleeding (GIB) – composite | K92.0, K92.1, K92.2, K28.0, K28.4 | ICD-10-CM |
| Secondary Outcome | Red blood cell transfusion | 30233N0, 30233N1, 30230N1, 30243N0 | ICD-10-PCS |
| Secondary Outcome | Acute myocardial infarction | I21, I22 | ICD-10-CM |
| Secondary Outcome | Ischemic stroke / TIA | I63, G45 | ICD-10-CM |
| Secondary Outcome | All-cause mortality | Death record in TriNetX EHR | TriNetX |
| Secondary Outcome | Urinary tract infection (falsification endpoint) | N39.0 | ICD-10-CM |
